# Potential effects of nano-cellulose and nano-silica/polyvinyl alcohol nanocomposites in the strengthening of dyed paper manuscripts with madder: an experimental study

**DOI:** 10.1038/s41598-022-23907-1

**Published:** 2022-11-15

**Authors:** Mostafa Abdel-Hamied, Rushdya Rabee Ali Hassan, Mohamed Z. M. Salem, Toka Ashraf, Merihan Mohammed, Nariman Mahmoud, Yasmin Saad El-din, Sameh H. Ismail

**Affiliations:** 1 Department of Conservation, Faculty of Archaeology, Cairo University, P.O. 12613, Giza, Egypt; 2 Forestry and Wood Technology Department, Faculty of Agriculture (El-Shatby), Alexandria University, Alexandria, 21545 Egypt; 3 Faculty of Nanotechnology for Post Graduates, Cairo University, El-Sheikh Zayed, 6th October, Giza, 12588 Egypt

**Keywords:** Engineering, Materials science, Nanoscience and technology

## Abstract

In the present work, the composite cross-linked were used to consolidate the dyed paper manuscripts. Nanocomposites of mesoporous silica nanoparticle (MPSNP)/polyvinyl alcohol (PVA) and cellulose nanofiber (CNF)/PVA, which have never been used before, have been evaluated for the consolidation process of the dyed paper manuscripts with madder extract. Three concentrations 1%, 3%, and 5% have been prepared. Analysis and investigation methods like scanning electron microscope (SEM), transmission electron microscope (TEM), dynamic light scattering analysis (DLS), X-Ray diffraction Analysis (XRD), atomic force microscope (AFM), Fourier transform infrared spectroscopy (FTIR) and total color difference (ΔE) by spectrophotometer have been used in order to characterize the prepared nano-sized composites and evaluate the treated dyed paper samples before and after the aging process. The results of surface morphology by SEM revealed the effectiveness of MPSNP/PVA core–shell nanocomposite at 5% in the consolidation process, where the improvement of properties of the aged dyed paper samples. The fibers of the treated paper became strong and appeared clearly. The result of ΔE measurements showed that the treated sample with MPSNP/PVA nanocomposite at 5% gave the lowest ΔE (5.22), while, the treated sample with CNF/PVA nanocomposite at 5% gave the highest ΔE value (11.66). Mechanical measurements (tensile strength and elongation) revealed the efficiency of MPSNP/PVA nanocomposite at 5% in the treatment of the aged dyed paper samples. The treated sample with the mentioned material gave tensile strength and elongation values of 84.8 N/nm^2^ and 1.736%, respectively. In contrast, the treated sample with CNF/PVA nanocomposite at 1% gave the lowest tensile strength and elongation values 38.2 N/nm^2^, and 1.166%, respectively. FTIR analysis revealed an increase was noticed in the CH_2_ stretching band (refers to the crystallinity of cellulose), where the intensity of the treated sample with MPSNP/PVA nanocomposite was at a 5% increase compared to the control sample. The FTIR results supported the results of mechanical measurements. The intensity of the CH_2_ stretching band, which refers to the crystallinity index of cellulose, was increased with the use of MPSNP/PVA nanocomposite at 3% and 5%, which explains the improvement in mechanical properties. This may be due to the nano-mineral particles, which improve the mechanical properties. Additionally, they reduce the effect of accelerated thermal aging on the cellulosic fibers and give them stability. The detailed analysis of analytical methods used for evaluation revealed the novelty of MPSNP/PVA nanocomposite, especially at 5%. It has a potential role in strengthening and improving different properties of the dyed paper manuscripts with madder extract.

## Introduction

Several libraries, museums, archives, and stores contain different dyed paper manuscripts. Paper is one of the most common materials used to transfer knowledge and cultural heritage^1^, whereas paper manuscripts are an essential part of the cultural and economic progress of humanity^2^. Paper is essentially composed of cellulose fibers, which are constituted of repeating β-d-glucopyranose units bonded together by a β-glycosidic bond to form a highly ordered structure^1,3^. The formed long chain is connected to additional hydrogen bond chains that give paper fibers their strength^3^. Fine bundles of cellulose molecules are formed, which are then joined to form fibers^3^.

Dye is defined as a color additive material able to transmit its color to substrates such as paper from chemical interaction with substrates^4^. Natural dyes are extensively used in dying historical paper manuscripts^5^. Dyes were used in Persian manuscripts in order to dye the paper support and to give some shades to specific details in miniatures^5^. Madder (*Rubia tinctorum*) is one of the oldest and most frequently natural dyes used in traditional dyestuffs known to man^6^. Additionally, madder was the main vegetable red dye in ancient Egypt^7,8^, where *R. tinctorum* was cultivated and used as far back as the Bronze Age^6^. Alizarin is considered to be the major dyeing component in the madder plant and effective dyeing species in madder^9–11^. Madder extract was used to dye cotton, linen, wool, and silk and the HPLC analysis of the extract showed the presence of salicylic acid, quercetin, ellagic, and benzoic acid as the main compounds^12^.

Unfortunately, natural dyes are less light stability and less permanent^5^, especially in uncontrolled environmental conditions. There are many dyed paper manuscripts in museums, libraries and archives that suffer from unsuitable environmental conditions that can lead to brittleness^13^.

Embrittlement is considered one of the most common signs of deterioration for dyed and non-dyed paper manuscript collections caused by factors that lead to the weakness of these manuscripts^14^. There are more reasons that lead to the embrittlement of paper, specially dyed with sensitive natural dyes. Paper is an organic material and unfortunately, it is under the action of many degradation factors: physical, chemical, biological, or social lead to its embrittlement and other signs of deterioration such as fungi growth, attacked by insects, losing the sizing agent, and other^2,13,15^. The lignocellulosic composition of paper is a suitable material for microbial growth, causing irreversible damage to paper manuscripts and artifacts^1^. Historical papers often have endogenously degraded paper areas^15^. The oxidation of additives used during the paper-making process can act as catalysts for acidic hydrolysis^1^. Paper made of low quality can lead to oxidation, which damages the paper fibers^16^.

The usage of alum-rosin sizing in paper-making has been identified as the main reason for the degradation of paper^17^. That is because the standard aluminum compounds used in paper-making hydrolyze with the release of acidity, and acidic conditions are recognized to promote degradation of cellulose, decreasing its degree of polymerization^18^.

The natural lignin in the paper composition, accelerates the rate of cellulose disintegration, especially in sunlight or industrial light, causing the paper to yellow, fade or darken due to the absorption of harmful UV rays^19,20^. Moreover, it also causes dehydration and brittleness^21^. Acidity derived from different sources is one of the most important causes of paper fragility, where acidic hydrolysis glycosidic bond scission inside the fibers occurs, producing a significant decrease in paper mechanical properties^1^. Further, polluted atmospheres contain acid, which can entire the fiber with low-density, and amorphous regions resulting in cutting the polymer chain via hydrolysis^18^.

Aging processes in paper cause yellowing and loss of strength, in which the most prominent response is the hydrolytic degradation of the cellulose macro-molecules^22,23^. The accelerated aging of papers of pure cellulose with low initial pH and degree of polymerization affects embrittlement, with a considerable loss of paper strength^18^. The natural dyes are more sensitive to most treatment materials such as organic solvents, toluene, ethyl alcohol, and others, which lead to the fragility of the treated paper manuscripts^24^. Inappropriate temperature, light, relative humidity (RH), air-pollutants and handling storage can lead to brittleness to paper materials^25^. Light leads to a great deal of damage for dyed manuscripts, where the light leads to yellowing and disappearance of some dyes and inscriptions and weakening of the paper manuscript’s components^3,26^.

Therefore, the conservation and restoration of dyed paper manuscripts refer to the series of operations taken to extend their lifetime by protecting them against deterioration factors, or by repairing the degradation they underwent^2,13^. Conservation treatments are necessary in order to preserve the degraded paper artworks to preserve their artistic and historical values and protect these paper manuscripts from deterioration, preserve the integrity of the paper, and increase its durability^1,27^. Accordingly, the consolidation of fragile dyed and non-dyed paper artworks is a necessary process^2,27^.

The paper manuscript is partially or totally covered with different consolidation materials, in order to achieve the following objectives; increase the mechanical properties of paper sheets, prevent ink bleeding on the historical paper's surface, give strength under acids action and at oil penetration, and decrease penetrating and sticky dust on historical paper surfaces^2^.

Some different materials have been previously used in the consolidation process of historical paper manuscripts. Cellulose derivatives such as sodium chloride of carboxy methyl cellulose (CMC), methylcellulose (MC)^28^ and hydroxy propyl cellulose (HPC)^29,30^ are yet the most common consolidants for paper manuscripts^2^. Additionally, several natural materials such as Funori have been used to consolidate paper manuscripts^31,32^.

Unfortunately, the consolidation process by cellulose ether materials has some disadvantages such as the poor improvement of the mechanical properties of treated paper^2^. The consolidation of paper by chitosan led to high color changes and a reduction of pH measurements for the treated paper^13^.

Keeping in mind all these facts and for avoiding the previous disadvantages, an innovative consolidation strategy for degraded historical dyed paper is presented based on the application of Nano composites. Composite materials are commonly preferred in different applications these days^33^. Nanocomposites display distinctive properties due to the nano-metric size effect, compared to conventional composites^34^. Hybrid materials are characterized by chemical stability and good abrasion resistance, where inorganic–organic hybrid materials can obtain properties that purely inorganic or organic materials cannot give^35^.

Nano-sized materials have become very common in the conservation of historical and cultural heritage, and numerous have been used for the consolidation of paper manuscripts and textiles^36–38^. Nano-crystalline celluloses are the ideal materials for the conservation and restoration of historical paper manuscripts, where nano-cellulose is characterized by a high affinity for the cellulose fibers as in paper because they are composed of the same biomaterial, cellulose^27^.

Nano-crystalline cellulose is characterized by nontoxicity and minimal environmental hazard, biocompatibility, biodegradability, huge surface area, optical transparency, high level of crystallinity around (54–88%), excellent stability and display of unique mechanical and thermal properties^1,27,33^. Additionally, cellulose nano-crystals are frequently used in the information of nano-composites, where they are extremely efficient in nano-composite formers^27^. Accordingly, this nanomaterial has different applications in numerous fields due to its unique properties^1,27^. Furthermore, they provide the most homogeneous layer, which effectively protects paper from mechanical changes caused by changes in relative humidity^27^. Consequently, nanocrystalline cellulose was chosen as the main material in the nanocomposites of this study for consolidation of the historical dyed paper manuscripts^15,37^.

Colloidal nano-silica is characterized by non-toxicity, cheapness, easily, and largely availability^39^. Additionally, it has consolidating properties for paper together with alkaline nature^39^. Moreover, nano-silica (SiO_2_) has been effectively used in the consolidation process of painting and pigments^40^.

PVA reinforced with nano-silica and nano-cellulose nanocomposites were synthesized by Ching et al.^41^ and increased in tensile strength primarily due to added nanocellulose and the mobility restriction of the PVA/nanocellulose composites due to the increase of nanosilica particles content. Tanpichai et al.^42^ mentioned that the addition of nano-cellulose increases the formation of hydrogen bonding within the paper, resulting in the improvement in the mechanical properties of the treated paper. Due to the characteristics of CNFs, they are used as reinforcement in composites and as CNF-polymer hybrid materials^43^.

Given the above-described advantages of nano-crystalline cellulose, nano-silica, and PVA materials, the use of some nanocomposites in conservation treatments of historical dyed paper artwork is presented, using nanocomposite from PVA with nano-cellulose and PVA with nano-silica.

## Materials and methods

**Figure 1:**
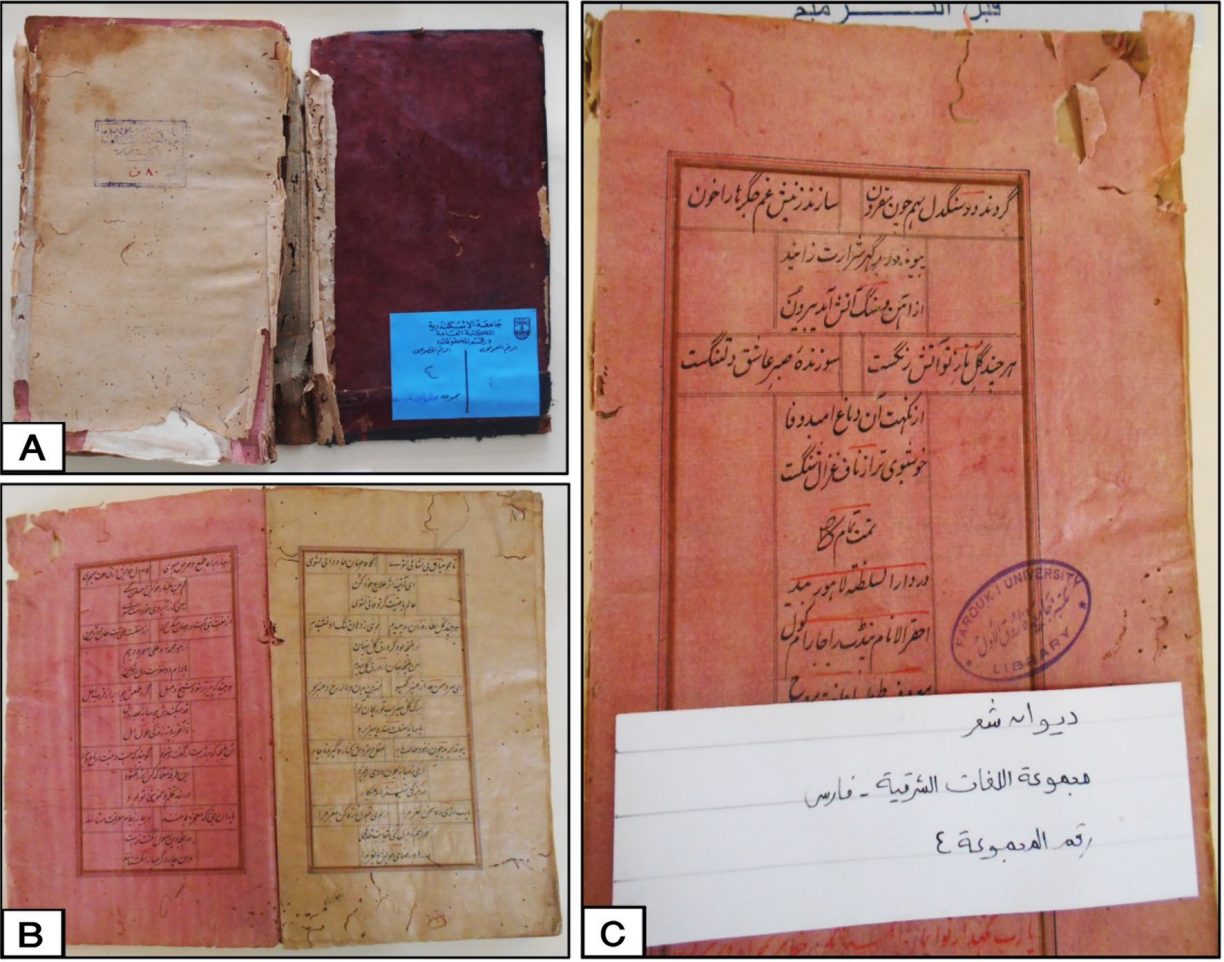
Deteriorated dyed paper manuscript at the central library of Alexandria University; (**A**) the paper manuscript with dyed leather bookbinding; (**B**,**C**) Some dyed paper sheets with madder and presence of different deterioration signs such as fading, tears, insect damage and other. Photos were taken by the co-author Mostafa Abdel-Hamied.

This study has complied with relevant institutional, national, and international guidelines and legislation. This study does not contain any studies with human participants or animals performed by any of the authors, where the survey study was carried out by the researchers on a deteriorated dyed manuscript entitled "DiwanShaar" (Fig. 1) returns to the poetry collection in Persian, which includes the Eastern-Persian language group. It's Public no. 4 and housed at the Central Library of Alexandria University, Egypt. The visual assessment for the studied manuscript revealed that this manuscript was dyed with madder dye and has different signs of deterioration, especially embrittlement and other as fading of the dye used, tears, and insect damage due to unsuitable conditions.

### Modern paper samples and madder dye

Whatman filter paper (100% of pure cotton fibers, no. 4 was Model quantitative filter Whatman ashless, grade 41 paper diameter 55 mm was used in this study. The roots of madder (*Rubia tinctorum*) were purchased from Harraz (Agricultural, Seeds Medicinal plant Company). The collection of madder roots has been done under the permission of the Department of Conservation, Faculty of Archaeology, Cairo University, Egypt.

### Extraction method of madder dye from dried roots

**Figure 2:**
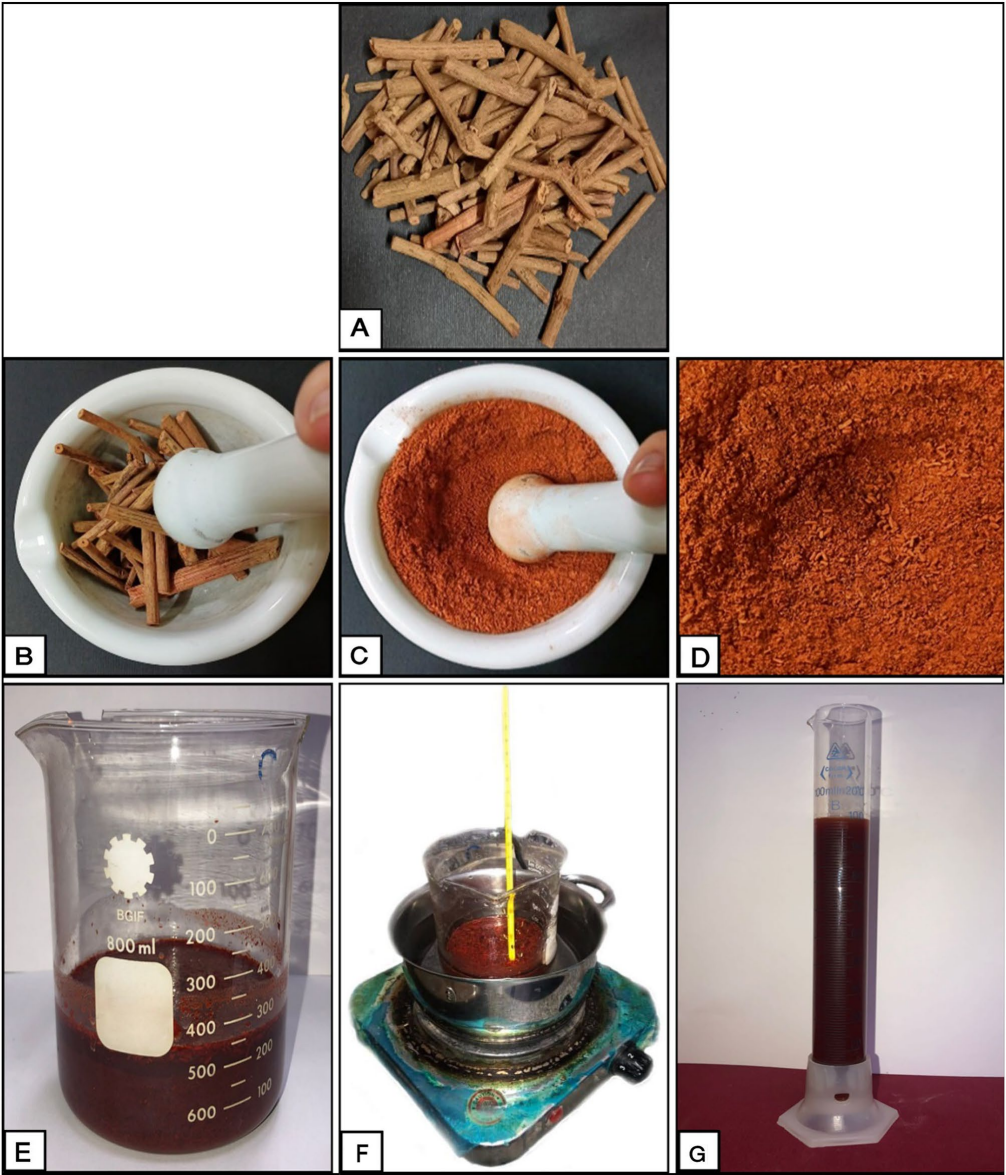
Extraction steps for the dried madder roots. The dried madder roots (**A**); The grinding process for the dried madder roots in porcelain mortar (**B**,**C**); The fine powder from madder roots (**D**); The final aqueous extract from the dried madder roots (**E**–**G**).

The extraction method of madder dye from the dried roots was performed^5,6^. Briefly, the non-essential materials have been removed by hand from the madder roots, then the dried madder roots (Fig. 2A) were excellently powdered down in pounding mills (porcelain mortar) until reaching the fine powder form (Fig. 2B–D). The ground madder powder was soaked in distilled water into glass beaker (Fig. 2E) for 24 h according to this ratio (40 g plant material: 800 ml distilled water). After soaking, the dyeing fluid was boiled in a water bath on a hotplate (Fig. 2F) for 1 h with stirring. This step aims to extract the dye from the madder solution. The temperature was measured during the extraction process and fixed at 70 °C. The dyeing liquor was left, in order to reduce temperature and reach room temperature. The dyeing liquor was filtered by Whatman Filter Paper in order to remove the insoluble residues. After filtration, the final dye extract (Fig. 2G) is ready for the dying process of paper samples.

### Dyeing process of paper

**Figure 3:**
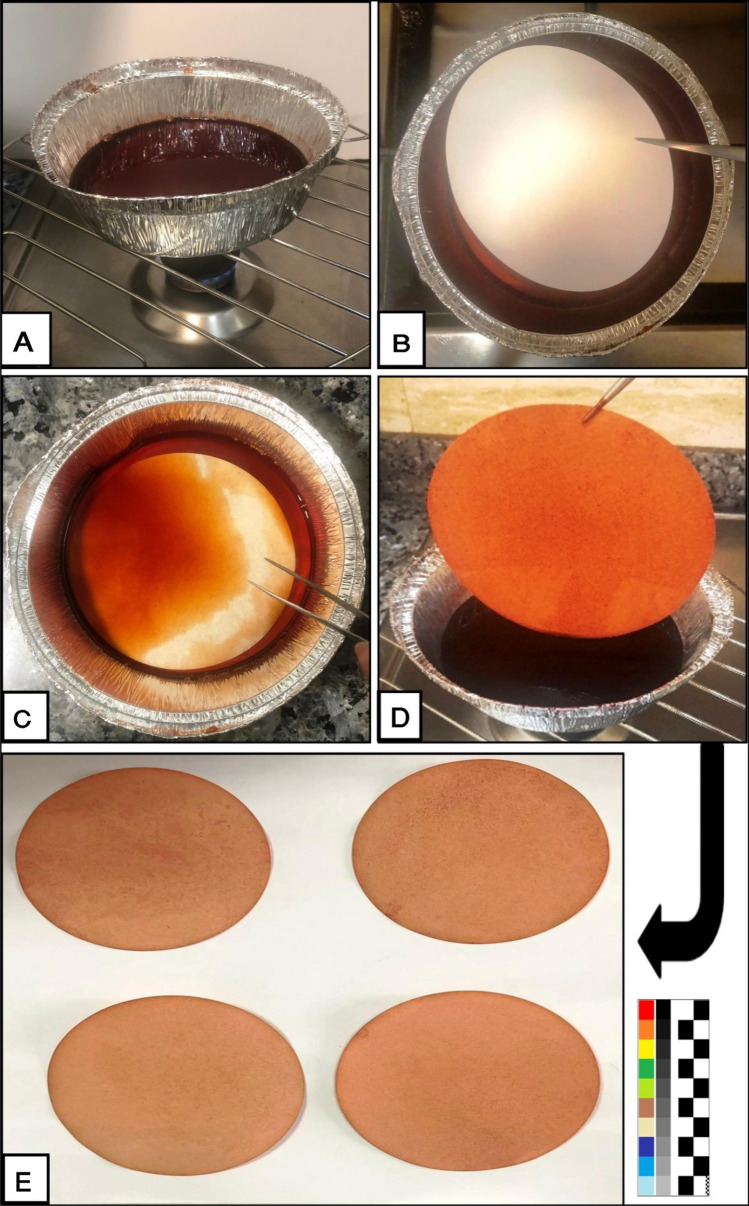
Dyeing process for Whatman paper; (**A**) The final aqueous madder dye in dye bath, (**B**,**C**) Immersion of paper sheets in the dye bath, (**D**) Removing of paper sheets from the dye bath by tweezers, (**E**) Drying of the dyed paper sheets in room temperature.

The dyeing process with madder dye for the paper was performed^6^. After preparation of the final aqueous madder dye, it was put in the dye bath (Fig. 3A). Alum solution (10%) as mordant was added to the dye at 60 °C for 30 min. Mordants such as aluminum and alum were often used to fix the colors of dye extract during dyeing and to improve the color fastness of dyes^5^. The paper sheet was immersed in the dye bath (Fig. 3B,C) and the heat was maintained at 70 °C for 1 h. In Islamic countries, the dying process of paper was usually performed by immersing prepared paper sheets in dye solution^5^. After a detected time, the dye extract was then allowed to cool down and paper sheets were removed from the dye bath using tweezers (Fig. 3D) and left for drying at room temperature (Fig. 3E).

### Preparation of nano-sized composites

#### Synthesis of cellulose nano-fiber (CNF)

The preparation of CNF was performed^44^ as follows: 50 g of cellulose of micron-size milling first in kitchen blinder for 10 min after soaking in water for 2 h then put air dry cellulose into stainless steel drum of 10 cm diameter. 250 g of silicon cribbed balls of 0.1 cm diameter were put in the same drum with cellulose and milling for 2 h at the speed of 5000 rpm using a ball mill instrument manufactured by photon company model of S220.

#### Synthesis of CNF/PVA nanocomposite

Synthesis of CNF/PVA nanocomposite is achieved in two steps; the first step is the synthesis of CNF by top-down techniques using the ball mill method. The second one is a hybrid polymer with CNF using the Sono-chemical method. 10 g of CNF added to 100 ml dissolved PVA in a beaker of 250 ml capacity and put into ultrasonic prop device manufactured by heister company model of S400 for 1 h at 60 kHz.

#### Synthesis of mesoporous silica nanoparticles (MPSNP)

MPSNP was synthesis by Vazquez and Inglesias method^45^ and Khalaf et al.^46^. Tetraethyl orthosilicate (TEOS) was used as an alkoxides precursor, while water and ethanol were used for hydrolysis of TEOS. In addition, cetyl tri ammonium bromide (CTAB) was used as a surfactant, and ammonia was used as a catalyst. However, a mixture of ethanol, doubled distilled water, ammonia and CTAB were stirred for 20 min, then a drop-wise of TEOS was added with a molar ratio of 1 TEOS:20 EtOH:45.6 H2O:10.4 NH4OH: 1 CTAB with continuous stirring for 4 h under room temperature until obtaining a white precipitate. Finally, the calcination process of the precipitate was set at 550 °C for 3 h.

#### Synthesis of MPSNP/PVA nanocomposite

MPSNP/PVA nanocomposite was synthesized by the sonochemical method. However, 1 g of prepared MPSNPs were added to 50 ml of PVA solvent in DMF and subject to ultrasound with the condition of cycle 0.4 and amplified 50% for 1 h until the homogenous white mixture was obtained, which air dry until white sheet obtain. Finally, the white sheet was milled using a ball mill for 2 h.

### Consolidation process of aged dyed paper sheets

**Figure 4:**
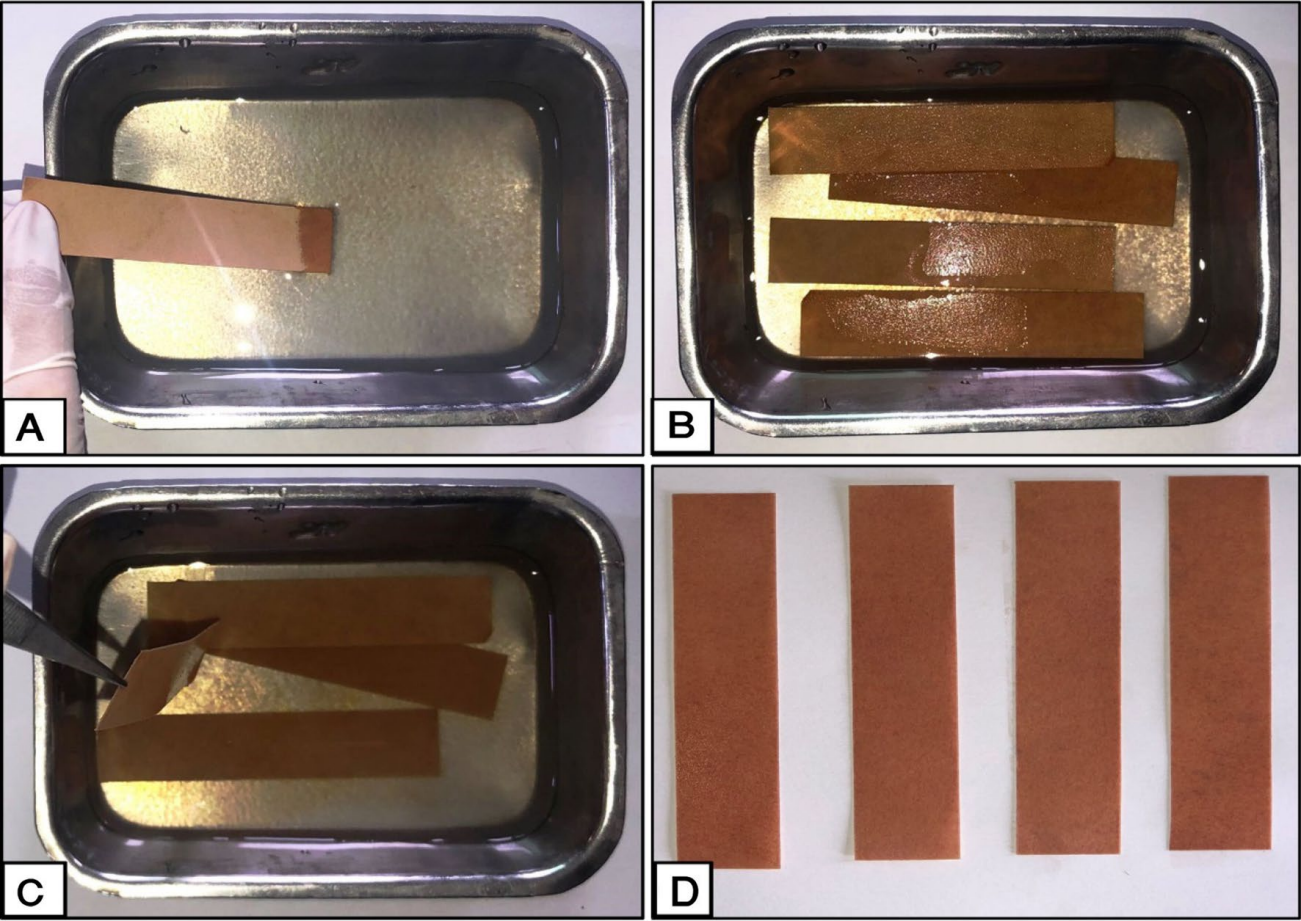
Consolidation process of aged dyed paper strips with prepared nanocomposites at different concentrations; (**A**,**B**) Immersion of the aged dyed strips in the prepared consolidants; (**C**) Removing the paper strips by tweezers after saturation and removing the excess from consolidants; (**D**) The treated paper strips before drying.

The dyed paper samples were consolidated by immersion technique in the prepared consolidation composites (Fig. 4) according to Rushdy et al.^13^ with three different concentrations of 1%, 3%, and 5%. The immersion method is commonly used in the consolidation process of experimental paper samples. The consolidation process was performed at the laboratory of the Conservation Department, Faculty of Archaeology, Cairo University, Giza, Egypt.

### Accelerated thermal ageing for dyed paper

The untreated and treated dyed paper samples were subjected to accelerate thermal ageing at 105 °C for 6 days according to Ardelean et al.^47^. In this process the oven (FN 500-Germany) at the laboratory of Conservation, Faculty of Archaeology, Cairo University, Giza, Egypt.

### Analytical techniques used

#### Scanning electron microscope (SEM)

Scanning electron microscopy (SEM) was used to visualize the composites nanoparticles and investigate their morphology. SEM images were taken using a Zeiss LEO Supra 55VP Field Emission and SEM Zeiss 1530. For sample preparation, composite nanoparticle suspensions were diluted 10 times with their dispersion medium, and then, a drop of the diluted nanoparticle suspension was directly deposited on a polished aluminum sample holder. Samples were dried under a vacuum. Samples were then coated in gold using EMITECH K450X sputter coater^48^.

On the other hand, the investigation of the surface morphology was performed on the aged un-treated, treated, and aged treated dyed paper samples by scanning electron microscope (SEM) in order to examine the changes in the surface morphology of the treated samples before and after artificial accelerated thermal aging and to evaluate the changes in the paper fibers which occurred as a result of the accelerated artificial aging process.

#### Transmission electron microscope (TEM)

TEM images were taken on TEM FEI Tecnai F20. For the measurements, the composite nanoparticle suspensions were diluted 10 times with their dispersion medium, and then, a drop was directly deposited on a 400-mesh copper grid and allowed to dry inside a desiccator for 24 h^48^.

#### Dynamic light scattering analysis (DLS)

Particle size measurements and size distribution of the composite nanoparticles were studied by dynamic light scattering technique (DLS) using a dynamic light scattering particle size analyzer Horiba LB-550 apparatus equipped with a diode laser with a wavelength of 650 nm and working at an angle of 90° and temperature 25 °C. The analysis was performed right after the preparation of the nanoparticle dispersions according to Ali et al.^48^. The zeta potential and size distribution of nano-TiO_2_ before and after modification were determined by the DLS method by using an SZ-100 Zetasizer (Malvern). Before, the samples were dispersed in distilled water according to Nguyen et al.^49^.

#### X-ray diffraction analysis (XRD)

XRD patterns were taken from the samples on a Siemens D5000 powder diffractometer (Germany) with 2*θ* in the range of 2°–80° and CuK*α* radiation (45 kV, 40 mA, *λ* = 1.5407° A)^49^.

#### Atomic force microscope (AFM)

The investigation of the surface topography of the prepared nanoparticles was achieved using a 2D and 3D Atomic Force Microscope at Faculty of Nanotechnology for Postgraduate Studies, Sheikh Zayed, Cairo University, Giza, Egypt. The technical information of AFM equipment are: AFM 5600LS, Agilent, Santa Clara, CA, USA. The sample preparation for AFM investigation was performed according to Elfaky et al.^50^.

#### Fourier transform infrared spectroscopy (FTIR)

The FTIR of the treated and aged treated dyed paper samples with nano-composites was done by Nicolet 380 FTIR Spectrometer device found at the National Institute of Standards (NIS), Haram, Giza, Egypt. The analysis was performed at a wavenumbers range from 400 to 4000 cm^−1^^51–53^.

#### Color change by spectrophotometer

The changes in the surface appearance of the paper samples before and after the aging process were determined by colorimetric measurements carried out by an Optimatch 3100^®^ from the SDL Company. The measurement was done according to Bergamonti et al.^1^ and Abdel-Maksoud et al.^54^, where at least 7 regions of a few mm^2^ in the area were examined and averaged on each paper sample, using five samples for each treatment to determine the total color difference ΔE^55^. It should be mentioned that all measurements were made before and after treatment compared to the control sample (aged un-treated dyed paper sample).

1 $$\Delta E=\sqrt{{( \Delta L)}^{2}+ {(\Delta a)}^{2}+{( \Delta b)}^{2}},$$

where, in CIELab notation, ΔL* is the change of lightness L*, Δa* and Δb* are the changes of the colorimetric coordinates a* and b* (red/green and yellow/blue opponent colors)^1^.

The colorimetric coordinates L*, a*, and b* of the CIE L*a*b* color space were used to express color change. The CIELAB color space is organized in cube form. The L* axis runs from top to bottom. The maximum for L* is 100, which represents white. The minimum for L* is zero, which represents black. The a* and b* axes have no specific numerical limits. Positive a* is red. Negative a* is green. Positive b* is yellow. Negative b* is blue. The effect of accelerated thermal aging for 7 days on the change in color parameters (L, a, and b) of untreated and treated dyed paper samples was studied.

#### Mechanical properties measurement

The mechanical properties (tensile strength and elongation) of the treated samples before and after the aging process were studied using the dynamometer produced by SDL ATLAS, H5KT at National Institute for Standards (NRC), Haram, Giza, Egypt. The measurement was performed with the following technical information: an average speed of 25 mm/min, the space between the device’s jaws was 5 cm; the width of the sample measured 3 cm and the length was 15 cm. An average of five measurements of each sample were taken for tensile strength and elongation. It should be mentioned that all measurements were made before and after aging compared to the control sample. It is the crunching force at which a strip of paper breaks off and is wide 1.5 cm, and the tensile strength in the longitudinal direction is greater than in the transverse direction, due to the arrangement of the fibers in the longitudinal direction with a density greater than the transverse direction.

### Statistical analysis

Results of mechanical and color changes were statistically analyzed with analysis of variance (ANOVA) using SAS software (SAS Institute, Release 8.02, Cary, North Carolina State University, Raleigh, NC, USA)^56^.

## Results and discussion

### Analytical techniques used for characterization of the prepared MPSNP/PVA nanocomposite

**Figure 5:**
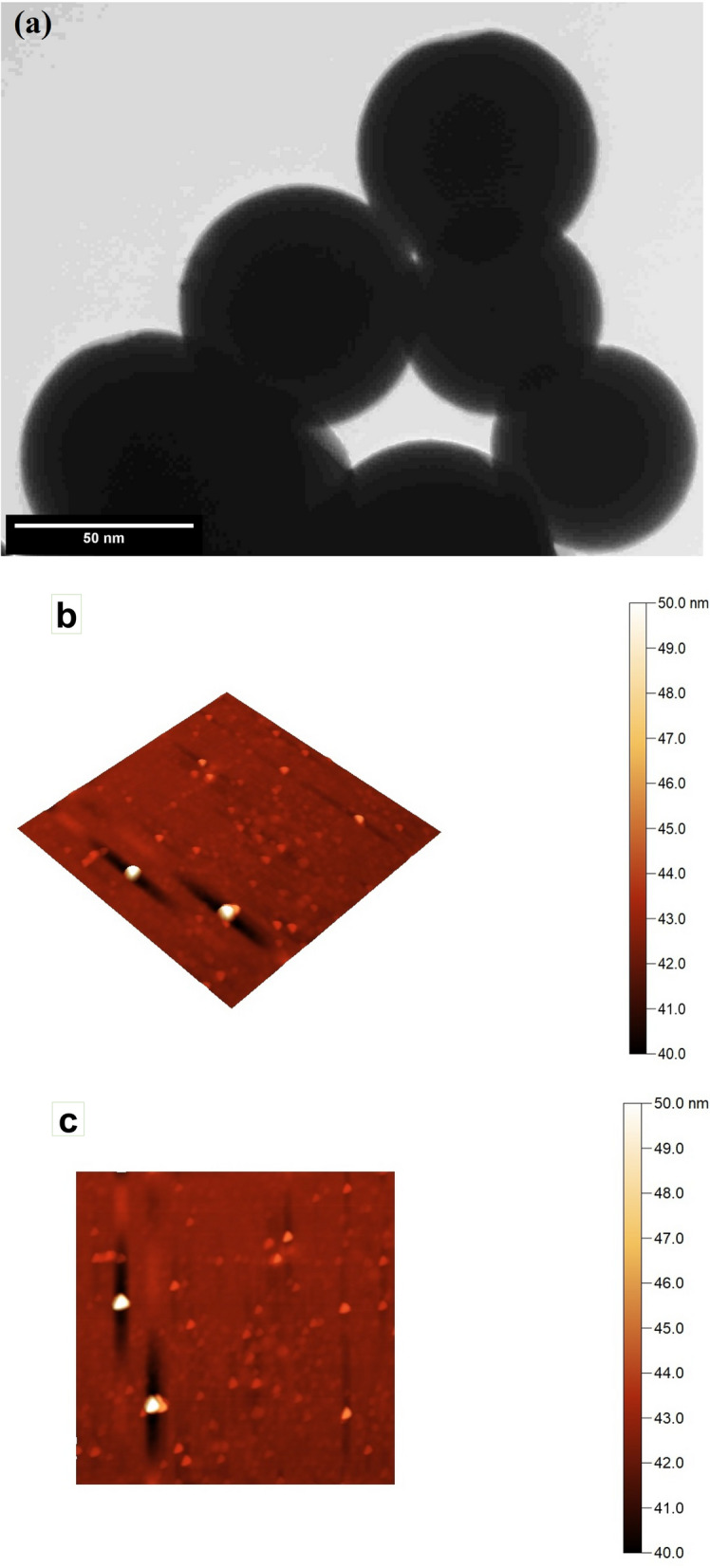

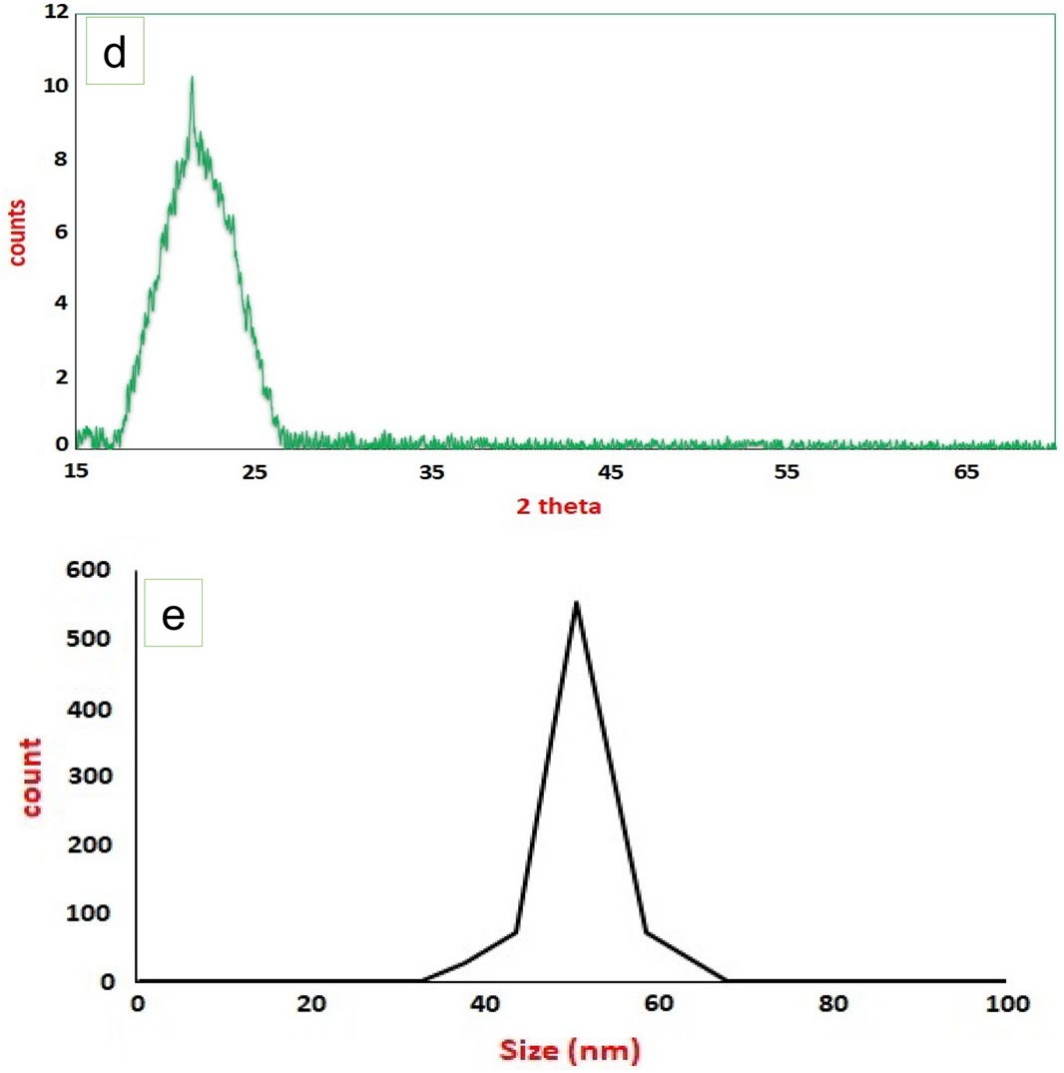
Analysis of MPSNP/PVA nanocomposite; (**a**) TEM image; (**b**,**c**) AFM images; (**d**) XRD pattern; (**e**) DLS.

Morphology characteristics by TEM (Fig. 5a) illustrate the spherical to sub-spherical shape with sharp edges of MPSNP/PVA nanocomposite with homogenous in shape and size. Additionally, the good desperation of the prepared composite nanostructure was noticed. AFM images (Fig. 5b,c) illustrate the spherical to sub-spherical shape with sharp edges of nano-composite with homogenous in shape and size. Identification characteristics of MPSNP/PVA nanostructure by XRD (Fig. 5d) illustrate the formation of the MPSNP/PVA nanostructure without any secondary products from synthesis methods and the amorphous nature of silica nanoparticles. The XRD pattern (Fig. 5d) showed the excellent purity of the prepared MPSNP/PVA nanocomposite, where the characteristic peaks of MPSNP/PVA nanocomposite was present without any secondary products from the synthesis method. The size of MPSNP/PVA nanocomposite characteristic by DLS (Fig. 5e) illustrates the size of MPSNP/PVA nanocomposite at about 50 nm which is compatible with TEM and AFM results.

### Analytical techniques used for characterization of the prepared NCF/PVA nano-composite

**Figure 6:**
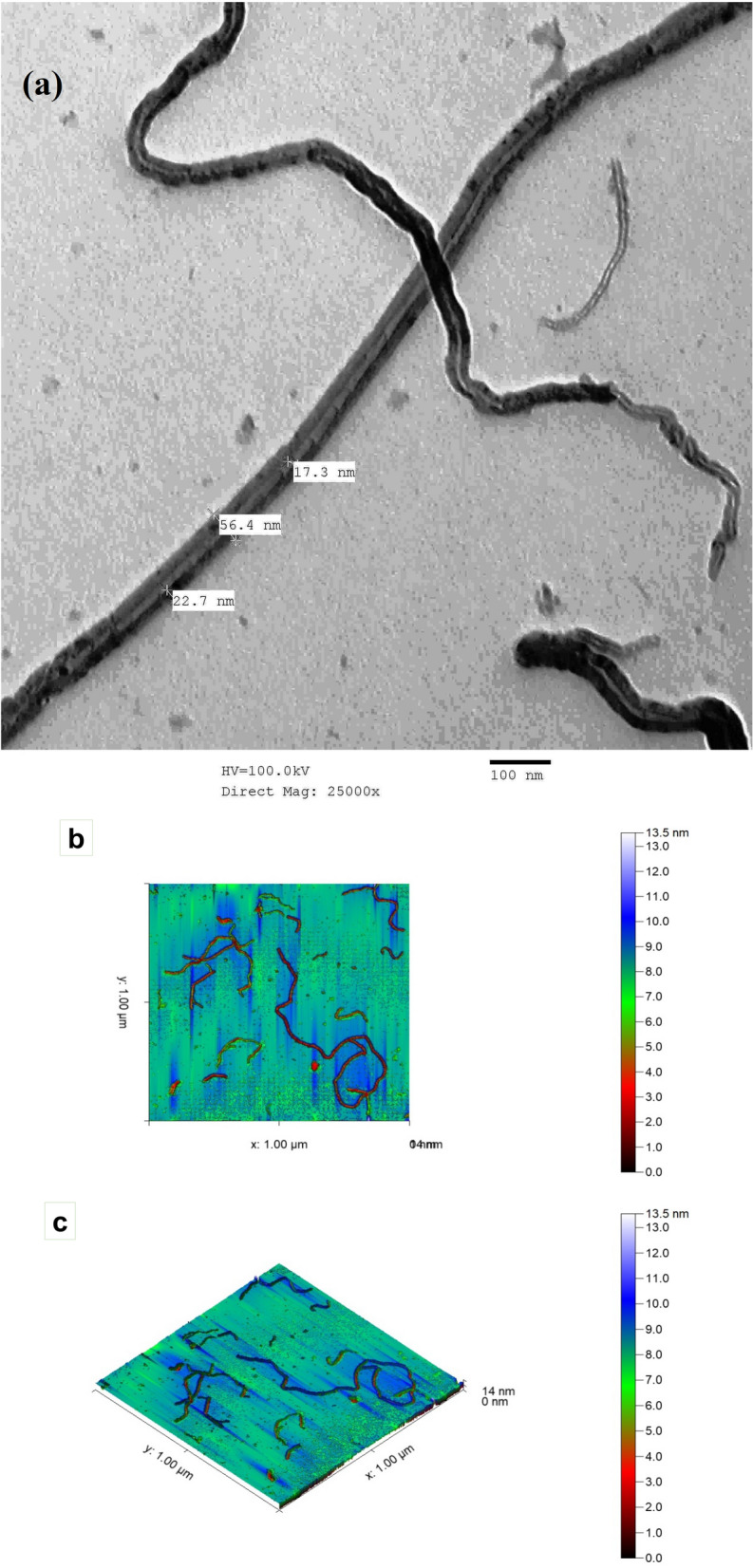

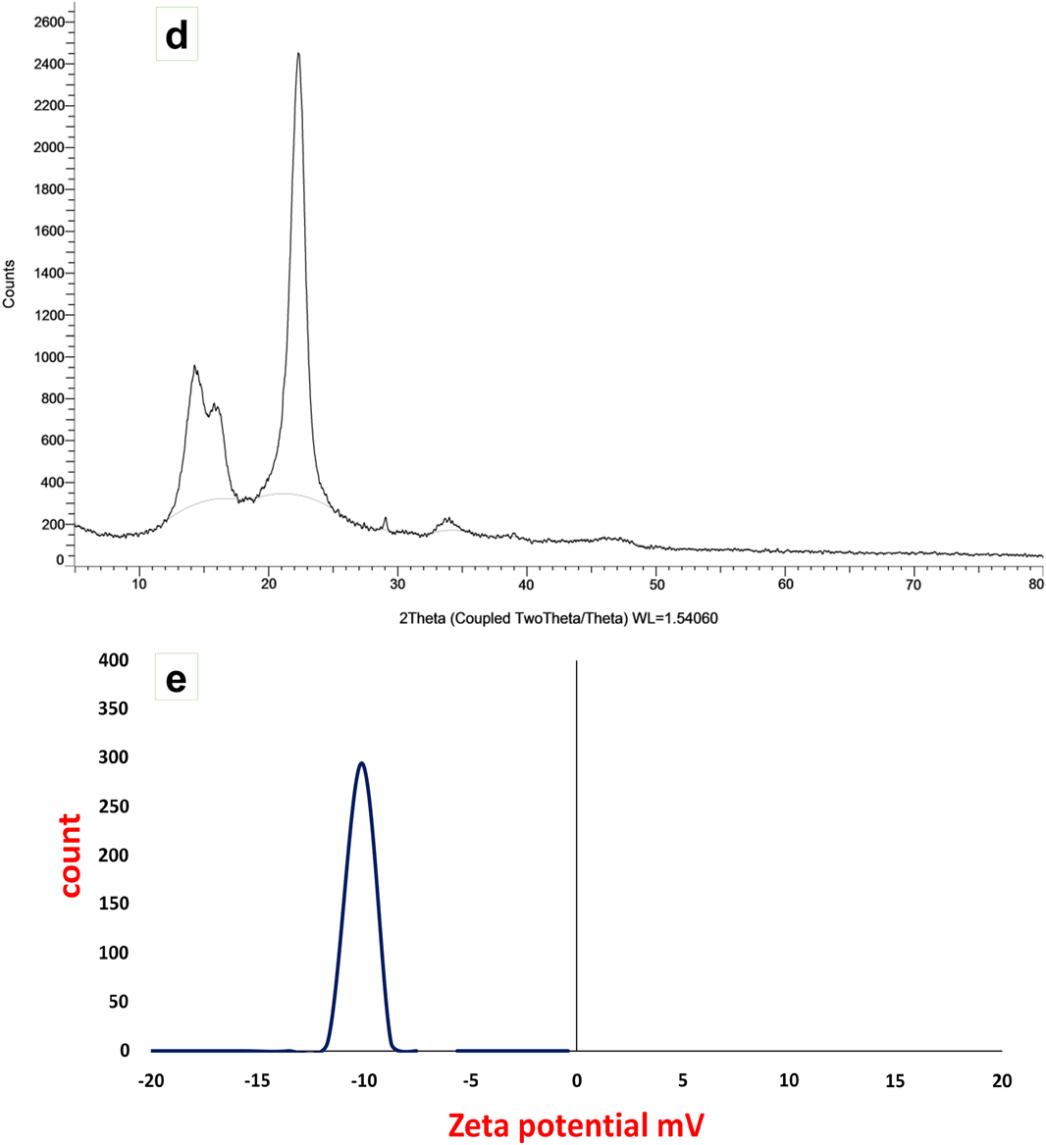
Analysis of NCF/PVA nanocomposite; (**a**) TEM image; (**b**,**c**) AFM images; (**d**) XRD pattern; (**e**) Zeta potential.

The image obtained from TEM (Fig. 6a) showed that the prepared nano-composite has the nano fibrous shape with a size of 17.3–56.4 nm. The AFM images, as shown in Fig. 6b,c, showed the nano fibrous shape of the prepared nano-composite, with a maximum height of 13.5 nm. Additionally, it was shown that a high degree of homogeneity of cellulose nanofiber. The XRD pattern (Fig. 6d) showed the characteristic peaks at 2*θ* = 14.3°, 15.8°, and 22.5°. The obtained results from XRD analysis revealed the high crystallinity of the prepared nano fibers. The Zeta potential of the prepared nano-composite illustrated in Fig. 6e, showed the high zeta potential value at − 10.1 mV.

### Analytical techniques used for evaluation of the untreated, treated and aged treated paper samples

#### Surface morphology of aged dyed paper samples by SEM

**Figure 7:**
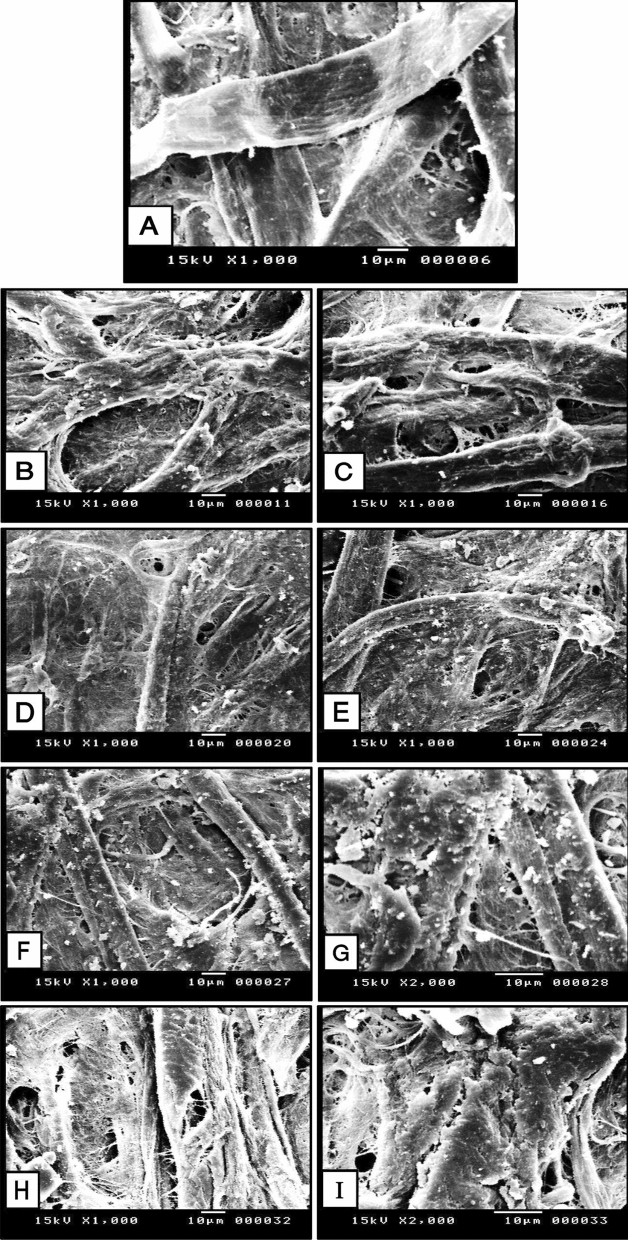
SEM micrographs of the untreated, treated, and aged treated samples with NCF/PVA nanocomposite; (**A**) aged untreated samples; (**B**) treated sample with 1% before aging; (**C**) aged treated sample with 1%; (**D**) treated sample with 3% before aging; (**E**) aged treated sample with 3%; (**F**) treated sample with 5% before aging; (**G**) accumulation of polymer on the paper surface; (**H**) aged treated sample at with 5%; (**I**) presence of cracks within the accumulated polymer on the surface.

Original cotton fibers have a diversity of distinct characteristics, the fibers are smooth with a twisted structure in addition to the parallel natural folds in the direction of the fibers. Scientifically, the typical diameter of cotton fiber ranges from 11 to 22 μm^57^. In contrast, after aging, the micro-fibrils of the primary wall were destroyed as shown in (Fig. 7A).

#### SEM for treated samples with NCF/PVA nanocomposite

The SEM image of the aged untreated dyed paper sample (1000×) (Fig. 7A) showed that the fibers appeared incoherent with gaps between them. This result is consistent with^58^ who noticed some deterioration with the aged untreated sample. We can also note that the fibers are relatively compressed, which may be the result of accelerated aging processes that reduce the proportion of physically adsorbed water on the walls of the cellulose fibers^59^.

The SEM micrograph (Fig. 7B) for the treated sample with nano-composite at 1% before aging showed a good material coverage and fiber coherence, and perhaps because of the low concentration, the consolidation material is penetrated into the cells of cellulose fibers and not just coated on the surface. The SEM micrograph (Fig. 7C) for the treated sample at 1% after aging revealed the separation of fibers due to the water loss and exposure to high temperatures under the impact of accelerated aging, which cause the fibers to separate and form gaps^57,58^.

Moreover, it was noticed that the thickness of the cellulose fibers increased with accelerated industrial aging. This may be attributed to the ability of the heat of aging to facilitate and speed up the movement of the reinforcement solution into the fibers, which led to an increase in the internal saturation of the fibers with the reinforcement solution^60^.

SEM of the treated sample with a concentration of 3% before aging (Fig. 7D) showed formed film apparently filling the pores among cellulose fibers^27^. Additionally, it was noticed from the SEM image of the treated sample at 3% that the nano-composite was absorbed by paper's fibers and appeared full from the inside, not external deposition which referred to the good penetration. Moreover, the fibers were more connected without the interstitial spaces. The SEM image (Fig. 7E) of the aged treated aged sample at 3% showed that the fibers started to separate slightly and also the presence of gaps.

SEM image (Fig. 7F,G) of the treated sample at 5% before aging showed the formation of a thin layer from the used nano-composite on the external surface. Additionally, the roughness of the surface was noticed. Moreover, the paper's fibers became hidden under the used consolidation material. The SEM micrographs (Fig. 7H,I) of the aged treated sample with nano-composite at 5% explained the behavior of treated dyed paper against thermal aging, where different gaps were observed with the paper and a weak cohesion with fibers was also noticed. Additionally, the presence of different kinds of cracks within the accumulated nano-composite layer was noticed.

It can be said that the investigation with SEM of the cotton paper samples treated with consolidation materials has shown an improvement in the surface morphology of the cellulose fibers in comparison with the control sample.

#### SEM for treated samples with MPSNP/PVA nanocomposite

**Figure 8:**
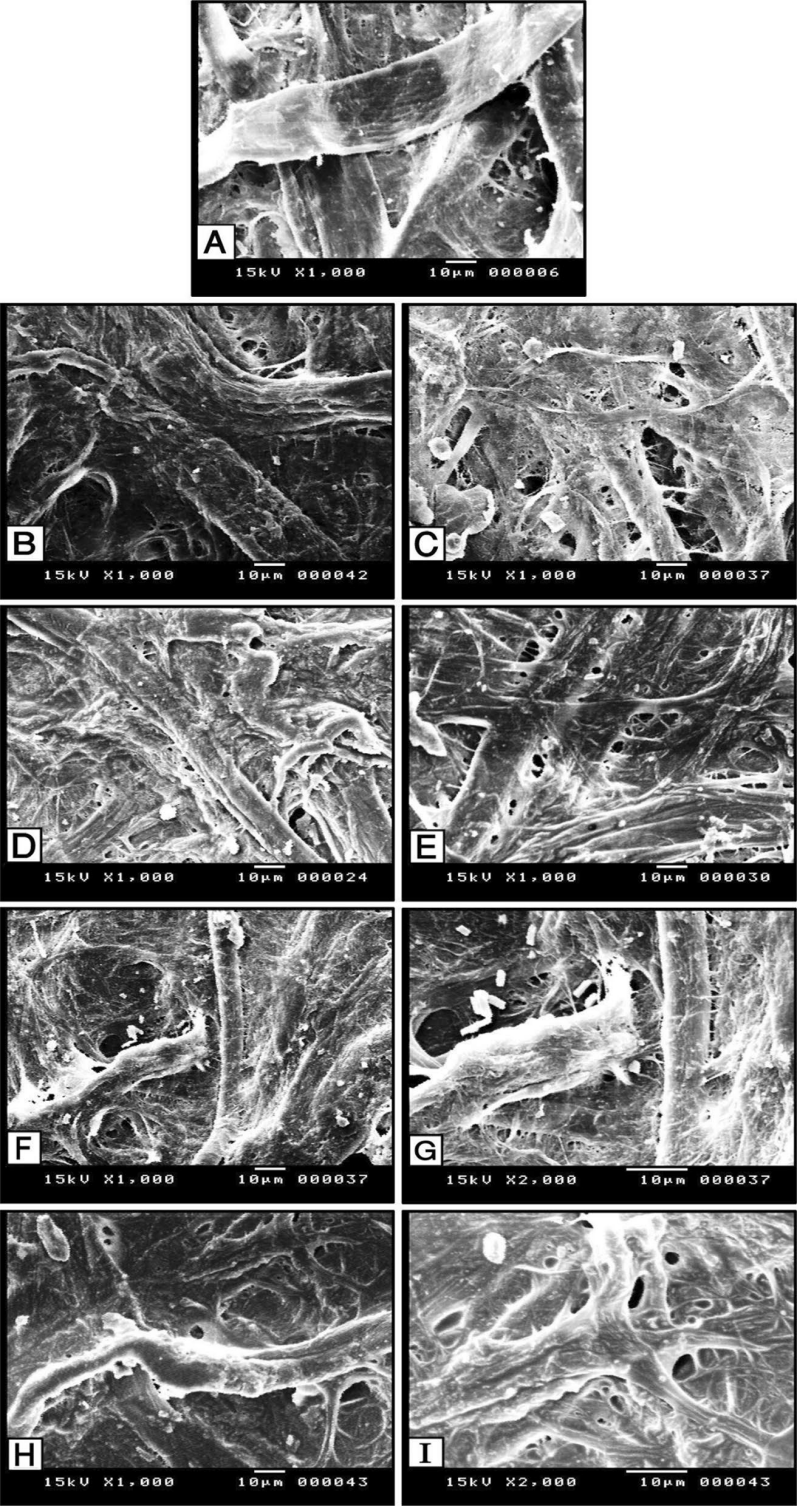
SEM micrographs of the untreated, treated, and aged treated samples with MPSNP/PVA nanocomposite; (**A**) aged untreated samples; (**B**) treated sample 1% before aging; (**C**) aged treated sample at 1%; (**D**) treated sample 3% before aging; (**E**) aged treated sample at 3%; (**F**,**G**) treated sample 5% before aging; (**H**,**I**) aged treated sample at 5%.

The SEM image of the aged untreated dyed paper sample (1000×) is shown in Fig. 8A). The treated sample with the prepared MPSNP/PVA nanocomposite at 1% before aging (Fig. 8B) showed that the fibers' coherence and good material coverage, as the fibers were supported due to the used nano-composite settled in some gaps causing an increased coherence of the fibers. SEM image of the treated sample at 1% after aging (Fig. 8C) showed some gaps and spaces between the fibers, which may be due to the exposure to high temperatures under thermal aging.

The SEM micrograph of the treated sample at 3% before aging (Fig. 8D) showed fiber coherence and good material coverage. The fibers were supported due to the settlement of the used nano-composite in some gaps, causing an increased coherence of fibers. SEM micrograph of the aged treated sample at 3% (Fig. 8E) showed the presence of paper fibers without any changes which referred to the resistance of this treated sample at 3%.

The SEM micrographs for the treated sample at 5% before aging (Fig. 8F,G) showed the presence of a thin layer on the outer paper surface which may be due to the high concentration and difficulty of the penetration inside the paper's fiber. Additionally, from the SEM micrograph, it was noticed the crystal shape of silica. The SEM images of the treated sample at 5% after aging (Fig. 8H,I) showed the penetration of the reinforced material into the paper, which helped to improve the appearance of the surface and fill the gaps. This may be due to the heat of aging by increasing the interaction between the cellulose fibers and the treated material.

### Measurement of color change

For the *L* value, the data obtained in Table 1 showed that the aged dyed treated sample with CNF/PVA nanocomposite at 5% gave the highest value (82.04), while the lowest value (74.64) was obtained from the treated sample with MPSNP/PVA nanocomposite at 5%. Table 1 refers to the artificial aging of treated paper sample CNF/PVA nanocomposite at 5% the lightness index increased (82.04), which confirms more lightness of dyed paper. On the other hand, the lowest percentage (74.64) was obtained from the treated sample with MPSNP/PVA nanocomposite at 5% as shown in Table 1.

**Table 1:** The results of color change for the aged dyed, treated and aged treated paper samples with CNF/PVA and MPSNP/PVA nanocomposites at different concentrations.

Nanocomposite treatment	Sample	*L*	*a*	*b*	*ΔE*
ADBT		69.71	12.22	18.77	0.0 m
CNF/PVA (1%)	TD	78.05	10.69	15.38	9.13f ± 0.015
	ADT	79.05	9.94	16.53	9.87e ± 0.015
CNF/PVA (3%)	TD	76.22	11.56	15.86	7.16k ± 0.015
	ADT	76.30	11.38	16.01	7.19j ± 0.015
CNF/PVA (5%)	TD	79.53	9.25	13.23	11.66c ± 0.015
	ADT	82.04	7.49	14.20	13.97a ± 0.015
MPSNP/PVA (1%)	TD	79.32	8.53	18.50	10.30d ± 0.015
	ADT	80.96	8.31	17.45	11.98b ± 0.015
MPSNP/PVA (3%)	TD	77.39	9.37	18.01	8.23 h ± 0.015
	ADT	77.98	10.05	17.34	8.67 g ± 0.015
MPSNP/PVA (5%)	TD	74.64	10.52	19.03	5.22 l ± 0.015
	ADT	76.48	9.35	18.84	7.35i ± 0.015

*ADBT* Aged dyed sample before treatment, *TD* treated dyed sample, *ATD* aged treated sample.

*Values are mean ± SD; means with the same letter within the same column are not significantly difference according to LSD at 0.05 level of probability.

**Table 2:** Mechanical properties (tensile strength and elongation %) for the aged untreated dyed paper, treated dyed paper and aged treated dyed paper samples.

Nanocomposite treatment	Sample	Tensile strength (N/mm^2^)	Elongation (%)
ADBT		35.2k* ± 0.158	0.77j ± 0.001
CNF/PVA (1%)	TD	38.2i ± 0.223	1.16g ± 0.041
	ADT	36.2j ± 0.291	1.09i ± 0.022
CNF/PVA (3%)	TD	42.4e ± 1.129	1.23f ± 0.015
	ADT	38.0i ± 0.406	1.12h ± 0.015
CNF/PVA (5%)	TD	54.5c ± 0.316	1.37d ± 0.015
	ADT	42.5e ± 0.430	1.26e ± 0.015
MPSNP/PVA (1%)	TD	41.7f ± 0.500	1.24ef ± 0.022
	ADT	39.5h ± 0.495	1.12h ± 0.025
MPSNP/PVA (3%)	TD	44.5d ± 0.524	1.47c ± 0.015
	ADT	40.8g ± 0.500	1.39d ± 0.015
MPSNP/PVA (5%)	TD	84.2a ± 0.255	1.73a ± 0.015
	ADT	79.7b ± 0.547	1.52b ± 0.015

*ADBT* Aged dyed sample before treatment, *TD* treated dyed sample, *ATD* aged treated sample.

*Values are mean ± SD; means with the same letter/s within the same column are not significantly difference according to LSD at 0.05 level of probability.

For the *a* value, the data obtained in Table 1, noticed that the aged dyed treated paper sample with CNF/PVA nanocomposite at 5% gave the lowest value (7.49), which means that there is an effect of this substance on the dyed paper over time compared to the treated sample before treatment (12.22). In contrast, the treated sample with CNF/PVA nanocomposite at 3% gave the highest value (11.56).

For the *b* value, the treated paper sample treated with CNF/PVA nanocomposite at 5% gave the lowest value (13.23). The highest value (19.03) was obtained from the treated paper sample with MPSNP/PVA nanocomposite at 5%.

For the total color differences (ΔE), the data obtained in Table 1 show the change in ΔE of all aged treated paper samples in different series as a result of heat aging. A slight difference in the ΔE value between the treated dyed paper and aged treated paper samples was observed. This result may be due to the durability of cellulose under heat aging. As the unit of cellulose has groups that absorb heat, the internal, and external chromophoric impurities or additives are able to induce its thermal degradation^61–63^. The thermal-degradation processes involved in cellulose are main chain scission, de-hydroxylation, dehydromethylation, and dehydrogenation, which resulted in to form of several free radicals that cause the degradation and yellowing of cellulose^64–67^.

The aged treated sample with CNF/PVA nanocomposite at 5% gave the highest ΔE (13.97). This result referred to the low resistance against accelerated thermal aging. Through the results, we find that paper coated with MPSNP/PVA nanocomposite (1%, 3%, and 5%) showed similar ΔE values before and after thermal ageing, but the 5% concentration was the best of them relative where it recorded a color change value 7.35. After aging, the latter total color change is less than strong perceptible differences. Therefore, we can conclude that the addition of MPSNP to PVA material contributed to the enhancement of resistance against accelerated thermal aging compared to CNF.

Previously, it mentioned that the relationship between ΔE and degree of color change was divided in to ΔE < 0.5 (very small difference), ΔE < 2 (small difference), ΔE < 3 (very perceptible difference), ΔE < 6 (perceptible difference), ΔE < 12 (strong difference) and ΔE > 12 (different color)^68,69^. According to the previous relationship, it can be said that the acceptable ΔE value (5.22) in the current study was obtained from the treated sample with MPSNP/PVA nanocomposite at 5% before aging. In contrast, the two nano-composites treatments, especially at 1% gave a strong difference in color (∆E < 12).

### Measurement of mechanical properties (tensile and elongation)

Table 2 shows that the mechanical strengths of the treated paper samples were highly affected by the treatment with both mixtures MPSNP or CNF/PVA nanocomposites at all concentrations. The data obtained (Table 2) show that incensement of the tensile strength of all treated paper samples with two nano-composites compared to the control sample (aged un-treated sample), which was recorded (35.2 N/mm^2^).

The treated sample with MPSNP/PVA nanocomposite at 5% before aging gave the highest tensile value (84.2 N/mm^2^). In contrast, the treated sample with CNF/PVA nanocomposite at 1% before aging gave the lowest value (38.2 N/mm^2^), where this treated sample recorded a slight increase in tensile value compared to the control sample (35.2 N/mm^2^). Additionally, the treated samples with CNF or MPSNP/PVA nanocomposites at 3% before aging gave a moderate increase in the tensile value. The decrease in tensile value for all treated samples with two prepared nanocomposites, but still, a good value compared to the value of the control untreated sample. The treated sample with MPSNP/PVA nanocomposite at 5% gave the highest value (79.7 N/mm^2^). This result referred to the good resistance against heat aging.

By comparing the previous data, it was found that the best result was obtained from the treated sample with MPSNP/PVA nanocomposite at 5%. Additionally, after aging, it became (79.7 N/mm^2^), which gives strength to fibers with aging. The dramatic increase of mechanical tensile can be attributed to the unique structure of mesoporous silica nanoparticles, which can function as an effective carrier for PVA, which contributed to the impregnation of the fibers with the PVA solution, which led to an increase in the tensile strength to high rates as compared to the results of CNF with PVA. Furthermore, the mesoporous silica nanoparticles are complex, consisting of organic templates (such as acetyl tri ammonium bromide) this leads to a rich variety of the construction of organized composites across PVA with complex morphologies of mesoporous silica^70^. Additionally, this improvement was actually attributed to the filling of voids between cellulose fibers by the consolidant used^71^.

An increase in the elongation value (Table 2) of all the treated samples was found compared to the control sample, which was recorded (0.77%). This result referred to the efficiency of all tested treatments in increasing the mechanical properties of aged paper, but the treated sample with MPSNP/PVA nanocomposite at 5% gave the highest value (1.736%). On the other hand, the treated sample with CNF/PVA nanocomposite at 1% gave the lowest elongation value (1.16%). The previous result showed the low efficiency of the treatment with CNF/PVA nanocomposite at 1%.

The previous results showed a positive linear relationship between both tensile strength and elongation; even after the aging, the mechanical properties of the aged treated paper were higher than those of the control (untreated) sample^72,73^. This means increasing the fiber bonding and consequently increasing the hydrogen bonding between fibers^69^. This result was confirmed by the FTIR analysis mentioned below. Scientists have attempted to improve and recompense for the mechanical properties that has been lost in deteriorated paper manuscripts by strengthening the hydrogen bonds between the weak fibers. In order to achieve this process, they used natural and synthetic polymers to improve the lost mechanical properties^57,71^.

### Fourier transform infrared analysis (FTIR)

In order to evaluate the treatment with the prepared nanocomposites in the consolidation process of dyed paper samples, the characteristic function group of paper must be illustrated. The characteristic function groups of paper are O–H stretching broadband; C-H stretching vibration band; C = O unconjugated band; C = O conjugated and H–O-H; CH2-COH and C-O, which were listed in Table 3.

**Table 3:** Characteristic function groups of paper.

Functional group	Wavenumbers (cm^−1^)	Intensity %	Characteristic
O–H stretching broadband	3334	90.9	Is characteristic for stretching vibration of the hydroxyl group in polysaccharides. This band includes inter-and intra-molecular hydrogen bond vibrations in cellulose
C–H stretching vibration band	2910; 2850	93.5; 93.9, respectively	Hydrocarbon constituent in polysaccharides
C=O unconjugated band	1739	91.9	
C=O conjugated and H–O–H	1624	96.6	Correspond to vibration of water molecules absorbed in cellulose
CH_2_–COH	1428	92.0	Associated with the amount of the crystalline structure of cellulose
C–O	1160; 1108; 1053; 1029	84, 80, 72 and 72, respectively	Cellulose polymerization

Data from Abdel-Maksoud and Khattab^74^.

#### Treated samples with CNF/PVA nanocomposite

**Figure 9:**
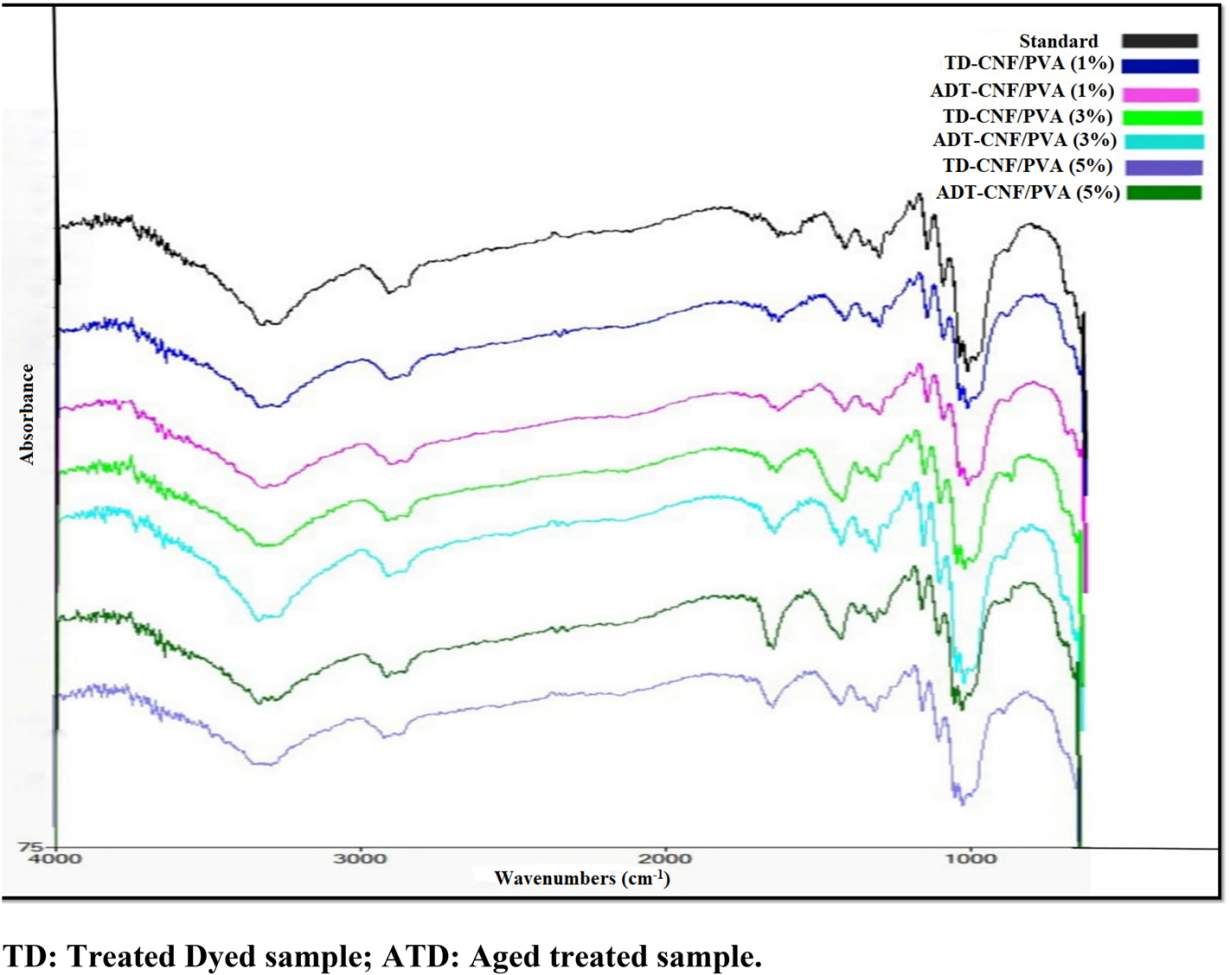
FTIR spectra of samples treated with CNF/PVA nanocomposite.

The FTIR spectra of the control sample (aged untreated sample) (Fig. 9) showed the oxidation process of this sample, where C=O group was noticed at around 1620–1664 cm^−1^ which referred to oxidation of the cellulose in the accelerated aging ovens^75^. The results of ATR-FTIR analysis (Table 4, Fig. 9) for the treated paper samples with CNF/PVA nanocomposite at different concentrations before and after accelerated thermal aging compared to the control sample revealed that treatment caused very slight changes which occurred in the cellulose water content, polymerization and crystallization levels, and these changes are summarized as follows:

**Table 4:** ATR-FTIR results of treated paper samples with CNF/PVA nanocomposite.

Function groups	Wavenumbers (cm^−1^)	Control	1%		3%		5%	
	Intensity		Before	After	Before	After	Before	After
OH stretching	Wave	3336–3266	3336–3269	3322–3255	3344–3261	3336–3263	3330–3258	3341–3266
	In	86.3–86.4	82.8	83.7–84.1	86.8–86.9	84.1–84.4	86.3–86.7	90.1–90.3
C–H stretching	Wave	2918	2910	2907	2915	2910	2915	2910
	In	88.7	85	85.7	88.8	87.7	88.6	92.4
C=O stretching	Wave	1694	1644	1641	1644	1646	1646	1649
	In	93.3	89.7	90.1	92.7	91.1	90.8	94.8
CH bending	Wave	1429	1427	1427	1427	1427	1427	1424
	In	92.4	89.6	90	90.4	90.1	91.5	95
Ch2	Wave	1314	1314	1314	1312	1314	1314	1314
	In	91.6	89.2	89.8	92.1	89.8	93	94.4
C–O–H bending	Wave	1162	1159	1159	1159	1159	1159	1159
	In	92.5	90	90.8	92.7	90.3	94.1	94.7
Non symmetrical in phase ring	Wave	1108	1105	1105	1103	1105	1105	1105
	In	89.4	88.3	89.3	90.4	87.2	91.8	92.1
C–O stretching	Wave	1028	1028	1025	1025	1028	1028	1028
	In	82.6	83	84.2	85.1	79.3	86	87.1
C–OH	Wave	661	661	664	664	664	661	653
	In	85.8	85.4	86.7	87.1	82.7	88.6	88.2

Before ageing: in the spectrum breadth of stretching O–H (3269–3336 cm^−1^), due to the effect of the used consolidation material on the cellulose water content. As it increased at the concentration of 3% before aging at 3344 cm^−1^, which indicated the occurrence of hydrolysis, because of the presence of hydroxyl groups in the structure of the treated materials. While it decreased at the 5% concentration before aging at 3330 cm^−1^.

From the cellulose supramolecular structure point of view, the spectrum part between 3700 and 3000 cm^−1^ (where the hydrogen bond formation could be observed) is from the most interesting spectrum parts^76^. According to the information presented in Fig. 9 and Table 4, the overall development of hydrogen bonding within cellulose of dyed paper samples has been investigated. The FTIR spectra of the treated paper sample at 3% (Fig. 9) shows a clear increase in O–H stretching broadening at 3344 cm^–1^, while a slight decrease in O–H stretching broadening in the treated sample at 5% at 3330 cm^−1^ before ageing was found. In the absorption intensity of the O–H stretching, a slight increase occurred in the treated sample at 3% which recorded 86.8%, while the control sample recorded 86.3%. This is may be due to the characteristic of NFC, where its addition to the nanocomposite led to formation of hydrogen bonding within paper^42^.

After ageing: by comparing the wavenumbers of the spectrum breadth of stretching O–H for all treated samples at different concentrations after ageing, it was observed that the wavenumber of O–H decreased in both concentrations at 1%, and 3% with 3322 cm^−1^, and 3336 cm^−1^, respectively. The slight decrease in the broadening of O–H stretching band confirms the breakdown of some of the hydrogen bonds^69,77^. On the other hand, the aged treated sample at 5% gave a slight increase with 3341 cm^−1^. The most noticeable change was in the 1% concentration after aging which recorded 3322 cm^−1^, which indicated a loss in water content due to thermal aging.

In the absorption intensity of the C–H stretching (2918 cm^−1^), due to the slight change in the water content of the paper cellulose, a slight decrease occurred in all the concentrations of the treated samples with CNF/PVA nanocomposite. In the absorption spectrum of the C=O stretching (1694 cm^−1^), which indicates the oxidation of cellulose, a slight decrease was noticed in all samples even after aging. Additionally, a very slight change in C–H bending (1429 cm^−1^) or C–O stretching (1028 cm^−1^)^58^.

#### Treated samples with MPSNP/PVA nanocomposite

**Figure 10:**
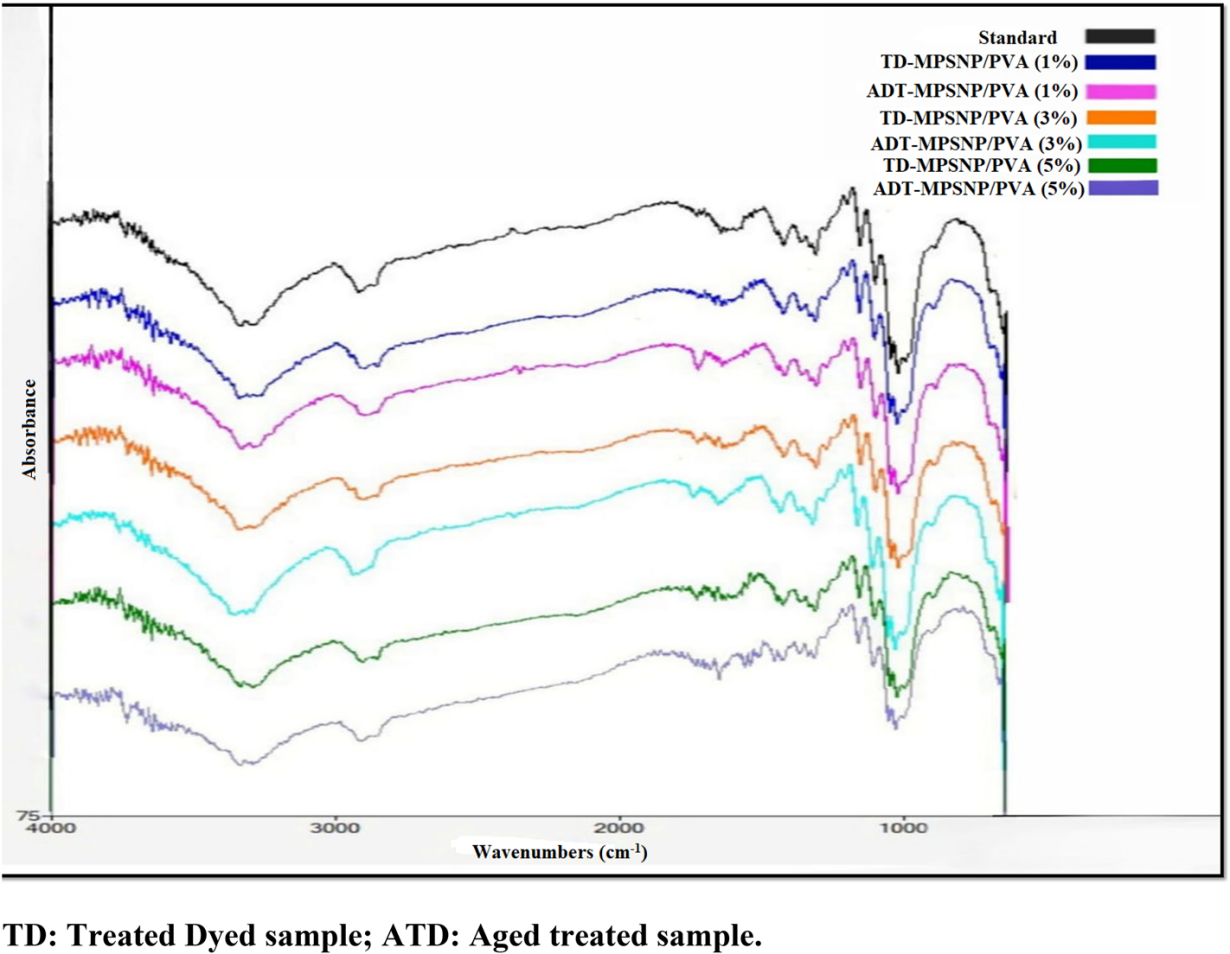
FTIR spectra of samples treated with MPSNP/PVA nanocomposite.

The data obtained (Table 5, Fig. 10) showed that O–H stretching we observe in concentration 1% before aging the intensity decreased is the result of loss of water molecules 82%. While after aging of 1% the intensity increased to be 86.9%, which indicates the occurrence of hydrolysis. With a concentration of 3% before aging, a slight loss in water molecules occurred. However, after aging, peaks moved to 3337 cm^−1^ and intensity increased to be 87.3%, which means there is hydrolysis. Before aging of 5%, the peaks moved to 3329 cm^−1^, and decreasing in the intensity is noticed 84.7%, which means a loss in water molecules. Also, it significantly decreased to 78.7% after aging.

**Table 5:** ATR-FTIR results of treated paper samples with MPSNP/PVA nanocomposite.

Function groups	Wavenumbers (cm^−1^)	Control	1%		3%		5%	
	Intensity		Before	After	Before	After	Before	After
OH stretching	Wave	3336–3266	3338–3261	3332–3277	3332–3267	3338–3267	3329–3280	3335–3273
	In	86.3–86.4	82–82.2	86.9–87	86.7–86.9	87.3–87.7	84.7–84.8	78.7–78.8
C–H stretching	Wave	2918	2901	2914	2907	2917	2901	2914
	In	88.7	84.3	89.5	89	90.4	86.7	80.6
C=O stretching	Wave	1694	1643	1643	1640	1646	1630	1650
	In	93.3	89.2	93.2	93	95.8	91.4	85.5
CH bending	Wave	1429	1426	1426	1426	1426	1423	1423
	In	92.4	88.5	92.5	92.3	95.2	91.2	86.7
Ch2	Wave	1314	1313	1313	1313	1313	1313	1313
	In	91.6	87.8	91.8	91.5	94.2	90.6	87
C–O–H bending	Wave	1162	1160	1160	1157	1157	1160	1160
	In	92.5	88.4	91.5	91.7	94.3	91.8	88.2
Non symmetrical in phase ring	Wave	1108	1105	1105	1105	1105	1105	1105
	In	89.4	86.6	89.3	89.7	90.9	90.1	86.3
C–O stretching	Wave	1028	1027	1027	1027	1027	1027	1027
	In	82.6	80.2	83.5	83.8	84.9	84	81.7
C–OH	Wave	661	661	661	661	661	661	661
	In	85.8	83.7	86.2	86.6	88.7	87.4	84.9

By comparing the wavenumber of O–H stretching for all treated samples at different concentrations before and after ageing, it was noticed an increase in the wavenumber for both treated samples at 3%, and 5%. The wavenumber of OH for the treated sample at 3% increased from 3335 cm^−1^ to 3337 and from 3329 cm^−1^ to 3338 cm^−1^ for the treated sample at 5%. This result confirmed the results obtained from mechanical properties, where the obtained result showed increase the fiber bonding and consequently increasing the mechanical properties. In contrast, the wavenumber of OH stretching for the treated sample at 1% decreased from 3338 cm^−1^ to 3332 cm^−1^. The decrease in the broadening of O–H stretching band for the treated sample at 1% referred to the breakdown of some of the hydrogen bonds^69,77^.

The C=O of 1% concentration before aging, the oxidation decreased (1643 cm^−1^, 89.2%). After aging the intensity slightly rebound to the standard sample (93.2%). In 3% before aging, the wavenumber is (1640 cm^−1^) and intensity slightly decreased (93%), which indicates the oxidation is less. However, after aging, the wavelength is (1646 cm^−1^) and the intensity increased to 95.8%, which means cellulose has been oxidized. Before aging of 5% concentration, the absorption moved to 1630 cm^−1^ and intensity decreased to became (91.4%). Furthermore, after aging, the beak became 1650 cm^−1^ and intensity significantly decreased compared to the control sample (85.5%), which indicates the oxidation will be less with aging.

The concentration of 1% before aging, its intensity in CH2 decreased (87.8%), while after aging the intensity slightly increased compared to the control sample (91.8%). In 3% concentration before aging, the intensity slightly decreased (91.5%), while after aging the intensity significantly increased (94.2%) that associated with an increase in the amount of crystalline structure of cellulose. The intensity of 5% before aging decreased (90.6%). Additionally, after aging decreases (87%), that means the crystallinity of cellulose will decrease with aging^74^.

## Conclusion

Herein, the consolidated materials were used as hybrid mixtures to improve dyed paper samples. As both nano-cellulose and nano-silica were mixed with PVA, different analytical methods were for the evaluation of the dyed paper before and after thermal aging. The results of the analytical techniques revealed that the treatment with the hybrid material, MPSNP/PVA nanocomposite at 5%, gave the best results in treating the dyed paper samples. Further, we recommend using MPSNP/PVA nanocomposite at 5% to consolidate the damaged dyed paper manuscript, where it proved its efficiency in the consolidation process.

## Data Availability

All data generated or analyzed during this study are included in this published article.
